# What’s new in chronic pain pathophysiology

**DOI:** 10.1080/24740527.2020.1752641

**Published:** 2020-12-30

**Authors:** Ivan Cohen, Mark J. Lema

**Affiliations:** Department of Anesthesiology, Jacobs School of Medicine, SUNY University at Buffalo and Biomedical Sciences, Buffalo, New York, USA

**Keywords:** pain pathophysiology, nerve growth factor, microglia, AMPK, genetics

## Abstract

The understanding of pain pathophysiology is continuously evolving. Identifying underlying cellular and subcellular pathways helps create opportunities for targeted therapies that may prove to be effective interventions. This article is an update on four areas of developing knowledge as it pertains to clinical management of patients with pain: nerve growth factor antagonists, microglial modulation, AMP-activated protein kinase activators, and genetic pain factors. Each of these areas represents novel targets for targeted therapies to prevent, treat, and modify the disease course of acute, chronic, and neuropathic pain. Currently most pain management techniques do not target these pathways directly, but there is promising evidence to suggest that the field is advancing toward available therapies in the near future.

## RÉSUMÉ

La compréhension de la pathophysiologie de la douleur est en constante évolution. Le fait de déterminer les voies cellulaires et sous-cellulaires sous-jacentes permet d’ouvrir des possibilités de traitements ciblés qui peuvent se montrer efficaces. Cet article fait le point sur quatre domaines du développement des connaissances relatives à la prise en charge clinique des patients souffrant de douleur : les antagonistes du facteur de croissance des nerfs, la modulation de la microglie, les activateurs de la protéine kinase activée par l'AMP et les facteurs génétiques de la douleur. Chacun de ces domaines représente de nouvelles cibles pour des thérapies ciblées visant à prévenir, traiter et modifier l'évolution de la douleur aiguë, chronique et neuropathique. Actuellement, la plupart des techniques de prise en charge de la douleur ne ciblent pas directement ces voies, mais il existe des données probantes prometteuses qui portent à croire que ce domaine progresse vers la disponibilité de traitements dans un avenir rapproché.

## Introduction

New discoveries in the mechanisms of persistent pain pathophysiology are evolving areas of research and developments of target-specific therapies. Conversely, the intricate interplay of multiple cellular and subcellular components and the ubiquitous presence of these components on non-nociceptive structures make treatments complicated by side effects.

Nonetheless, this review focuses on four evolving areas of pain research that show promise in effectively identifying and treating persistent pain. This is not an exhaustive list of ongoing research; these topics were selected to represent a cross section of various fields of study that enhance our understanding of pain physiology and have identified possible therapeutic targets. Though trials are underway in some of these areas, many are developmental and require further study before any agents are available for clinical use.

This article briefly introduces the state-of-the-art progression in nerve growth factor antagonists, microglia actions in the central nervous system, AMP-activated protein kinase (AMPK) activators, and genetic alterations affecting pain perception and treatment.

Further exploration of these topics can be reviewed by referring to the references included at the end of this article.

## Nerve Growth Factor Antagonists

Nerve growth factor (NGF) is a neurotropin implicated in inflammation and sensitization phenomenon. Increased concentrations have been found in tissues undergoing an inflammatory response. Biochemically, it is a polypeptide that binds to tropomyosin receptor kinase A (TrKA) and p75 receptors. TrKA receptors on mast cells trigger histamine release, potentiating the inflammatory pathway and eventual pain hypersensitivity. Activation of TrKA and P75 receptors on peripheral nociceptors results in downstream neurotransmitter cascade and increased transcription of various neurotransmitters and receptor proteins implicated in pain (substance P, calcitonin gene-related peptide, brain-derived neurotrophic factor, vanilloid receptor 1, etc.), which subsequently propagate pain signals to the central nervous system (CNS).^1^

It has been theorized that anti-NGF monoclonal antibodies might be able to intervene early in histamine activation and affect pain at the transcriptional level by decreasing production of cellular and subcellular elements critical to this pathway. Three antibody agents have been studied thus far. Tanezumab, a human immunoglobulin-2 NGF antibody, is being studied for osteoarthritis (OA), chronic low back pain, and metastatic bone lesions, among others. It has been found to be highly specific and selective for NGF, and in 2017 it was given fast-track status by the U.S. Food and Drug Administration. Meta-analysis of Phase 3 studies showed favorable outcomes versus placebo, naproxen, and oxycodone.^2^ Fasinumab is another agent in the final stages of trials for hip and knee OA. Fulranumab is a third agent that is no longer undergoing clinical investigation.

Unfortunately, there may be significant limitations when using these current agents. In 2010 all trials of these agents were put on hold due to concerns of rapidly progressive osteoarthritis. Trials were resumed in 2015, with the exception of fulranumab. The meta-analysis for tanezumab has revealed that doses of 10 mg or higher increase the risk of rapidly progressive osteoarthritis (as high as 6.3%), particularly with concomitant nonsteroidal anti-inflammatory use and a history of fracture.^2^ Subsequently, studies for tanezumab were limited to lower doses. Other common side effects include arthalgias, nasopharyngitis, and paresthesias.

Despite adverse effects, trials for tanezumab demonstrated analgesic benefit in patients with OA and low back pain if limited to 2.5 mg or 5 mg doses for up to 8 weeks.^1,2^ Further trials ranging from 16 to 56 weeks of therapy demonstrated similar results with less success in the 2.5 mg dose range. With mindful patient selection these agents may be indicated as an alternate line of therapy for those who have failed conventional regimes.

In summary, though there is promising initial data to support NGF antagonists as an analgesic modality, the current agents studied generally have side effects that outweigh the benefits, particularly at higher doses. It is hoped that dosing studies and further trials will elucidate a more favorable profile.

## Microglia and Neuropathic Pain

Microglia are cells of the reticuloendothelial system of the CNS. Arising from myeloid precursors derived from embryonic mesoderm, these cells migrate to the CNS and become microglia. They are responsible for shaping and reshaping neuronal circuits continuously throughout an entire life span. They comprise 10% to 20% of all CNS cells, with the highest densities in the hippocampus, basal ganglia, substantia gelatinosa, and spinal cord. They are responsible for a significant portion of what we call neuroplasticity and are involved in the cellular processes that take place during and after nerve injury, one of the pathognomonic features of neuropathic pain.^3–6^

When a nerve is injured, microglia undergo “microgliosis,” or activation and migration, which allows them to perform several responses, including protecting injured nerves, phagocytosis of cellular debris, and the release of cytokines and other inflammatory modulators to initiate and propagate an immune response. Persistent pain models have demonstrated that hyperalgesia is associated with microglial activation in the dorsal horn of the spinal cord.^4,5^

Microglial activation occurs through several pathways, perhaps most important the mitogen-activated protein kinase (MAPK) pathways.^3,4^ These systems affect intracellular transcription and play a critical role in inflammation. MAPK includes several signaling chemokines and cytokines, including extracellular regulated kinases, p38 protein, and c-Jun N-terminal kinase.^4^

Microglial p38 MAPK pathways are activated via phosphorylation by many proinflammatory cytokines and mediators, including tumor necrosis factor-α (TNF-α), interleukins (such as IL-1β), and adenosine triphosphate (ATP).^4^ These cytokines are released by the damaged afferent neurons themselves to activate microglial p38 pathways and ultimately induce transcription of further inflammatory mediators, including cyclooxygenase-2, brain-derived neurotropic factor, IL-6, TNF-α, and IL-1β. These mediators then activate surrounding astrocytes, increase recruitment of more microglia, and promote CNS sensitization and neuronal transmission. Glial TNF-α also causes increased synaptic efficiency for propagation of pain signals, increases endocytosis of gamma aminobutyric acid-A receptors, and increases N-methyl-d-aspartate receptor receptor activity through extracellular regulated kinase phosphorylation.^4^ This is effectively a downregulation of the inhibitory pain modulation response, resulting in increased pain signaling to thermal and mechanical nociception and hyperalgesia.

Regarding therapeutic potential, intrathecal administration of p38 inhibitors such as minocycline, a clinically used antibiotic, inhibits microglial activation and decreases pain behavior by attenuating neuropathic pain.^4,5^ Studies so far are limited to animal models, and few look at adverse effects. It is theorized that intrathecal administration would minimize systemic effects of minocycline, and one study did not find any evidence of neurotoxicity.^7^ Differential target multiplexed (DTM) spinal cord stimulation (SCS) has been studied in the setting of targeting spinal anatomy known to be richer in glial cells, particularly the T8–T11 region. Ongoing human studies targeting these regions with DTM SCS have produced preliminary results showing improved analgesic outcomes over conventional SCS for back and leg pain.^8^ It is thought that SCS affects glial cell interactions, which include intra- and intercellular communication, signal transduction, modified protein phosphorylation, and ion transport.^9^

Microglial influence on neuronal plasticity and the ability to modulate this response may have large implications in the treatment, prevention, and management of chronic pain syndromes. A better understanding and development of pharmaceutical and neuromodulatory interventions for these pathways may allow physicians to reduce the burden of chronic pain after nerve injury, including postoperative pain syndromes.

## AMPK Activators

AMPK is an enzyme involved in cellular homeostasis that maintains cellular energy levels by regulating ATP production and consumption.^10^ When a cellular stressor such as hypoxia, hypoglycemia, or chemical insult affects a cell, ATP levels decrease, which activates AMPK to restore the balance. AMPK also acts as an inhibitory regulator of several enzymatic pathways linked to chronic pain, including MAPK and mammalian target of rapamycin complex 1.^10,11^

Activation of AMPK can be achieved through several mechanisms. Direct allosteric activators protect the kinase from dephosphorylation via β or γ subunits.^10,11^ Indirect activators enhance phosphorylation at multiple sites thereby increasing enzymatic activity. Two known AMPK activators include metformin and O304. Metformin, a clinically used anti-diabetic agent, acts as an indirect AMPK activator nonspecifically through several intracellular sites and can cross the blood–brain barrier. Conversely, O304 does not cross the blood–brain barrier and is a specific AMPK activator that decreases phosphorylation of the Thr172 site on the alpha subunit via protein phosphatase 2 C and does so without suppressing that enzyme’s AMPK activating effects.^10,11^

Mouse model studies have demonstrated that AMPK activation can reduce peripheral nerve injury and allodynia within 7 days of injury.^12^ AMPK activators have also demonstrated complete resolution of long-standing nerve injury within 60 days of the event. One study looked at metformin and O304 in a postoperative pain mouse model and demonstrated reduced mechanical pain sensitivity with both agents compared with the drug vehicle. This study also demonstrated an even greater synergistic effect with both agents co-administered.^12^

There are rich opportunities for further study and expansion within this field. Metformin studies may be designed to evaluate treatment of postsurgical and neuropathic pain, possibly as part of a preemptive perioperative regimen such as an enhanced recovery after surgery protocol. There is also a possibility for further development and study of other allosteric AMPK activators such as A769662 and OSU-53 for clinical use.^10,11^ These agents are 100 times more potent than metformin, activating AMPK by targeting specific γ-2 subunits for both strong activation and avoidance of adverse effects.^10^ As specific allosteric agents, they also have a diminished “scatter” effect compared with nonspecific activators. These types of agents may represent a more robust therapeutic pathway compared with the currently studied drugs.

## Genetics and Pain Conditions

Genetic studies have revealed that nociception, chronic pain syndromes, and analgesic management all have links to heritable traits. Knowledge in this area is still expanding. This section will summarize a variety of genetic issues in pain management, including enzymatic activity, addiction, behavior, mutations, channelopathies, and specific heritable pain disorders.

Most currently available genetic testing emphasizes drug metabolism and pain response. One well-described system involves opioid metabolism by the cytochrome P450 family of isozymes. There are quantitative and qualitative interpersonal variations of this isozyme cohort, which can cause very different clinical outcomes during opioid therapy.^13^

A classic example is how CYP2D6 metabolizes codeine, an analgesically inactive prodrug, into morphine via O-demethylation.^14^ Roughly 7% to 10% of Caucasians, 1% of Asians, and 1.4% of African Americans are have a CYP2D6 deficiency rendering the drug ineffective.^14^ Some patients, including up to 10% of African Americans, have a genotypic variant for ultra-rapid metabolism^15^ that increases the rate of conversion to morphine, resulting in supratherapeutic drug levels that have adverse effects, including death. This heritable variability in enzymatic activity can clearly have very serious ramifications directly translatable to clinical practice and has led to an avoidance of these agents in pediatric populations after a number of reported drug-related fatalities.^16^

Other examples include μ-opioid receptor gene OPRM1. This receptor has a known polymorphism identified in cancer patients named A118 G with three genotypes (AA, AG, GG). Patients undergoing oxaliplatin chemotherapy with tramadol had differing analgesic outcomes based on allele genotype. The G allele is responsible for an amino acid change from asparagine to aspartic acid in the N-terminal of the μ-opioid receptor that may alter binding and/or response to substrates.^17,18^ The presence of the G allele resulted in high dose requirements and higher pain scores than AA genotypes.^18^ High frequencies of the A118G G allele variants have been found in opioid-dependent subjects of both Hispanic^17^ and Swedish^19^ origin.

Addictive behavior has also been identified as having a strong genetic association, with 43% of the variance in drug abuse due to genetic factors unique to opioid metabolism.^20^ The dopamine receptor has been a highly active target of study. Dopamine receptor D2 (DRD2) polymorphism is associated with addictive behavior; DRD2 gene Taql RLFP A (rs1800497) was studied in patients with an opioid use disorder versus control. Patients with either A1A1 or A1A2 alleles consumed twice as much heroin as those without the A1 allele.^21^ This allele is present in 19% of Caucasians with opioid dependence compared to 4.6% of those without drug abuse history.^22^ Dopamine D3 receptors are prevalent in the reward and reinforcement center of the nucleus accumbens. Opioid-dependent patients with high sensation-seeking scores are more likely to be homozygous for the DRD3 allele compared to patients with lower scores.^23^

There are other non-opioid-related genetic pathways implicated in acute and chronic pain syndromes. One area of developing knowledge involves channelopathies. This term refers to a loose organization of more than 30 heterogeneous conditions that all share the root cause of genetic defects in ion channel function. These defects may be inherited (i.e., heritable mutations in genes encoding the channel proteins themselves) or acquired (de novo mutations, drug/toxic effects, or autoimmune phenomena). Commonly recognized non-pain examples include cystic fibrosis (CFTR Cl^−^ channel), long QT syndrome types 1 and 2 (delayed K^+^ channel), long QT syndrome type 3 (Na^+^ channel), epilepsy (voltage-gated Ca^++^ channel), and diabetes mellitus (ATP-sensitive K^+^ channel).^24^

Numerous mutation sites may cause a similar end phenotype or disease state. For example, cystic fibrosis is a disease that may result from any one mutation of more than 1000 described variants in the CFTR gene. These mutations can be as small as a single nucleotide polymorphism (SNP) in which a single base-pair substitution occurs in the genetic code at a specific point in the genome. This base pair can then have a downstream effect on the gene expression, including qualitative and quantitative defects of the resulting protein complexes. SNPs that occur together with high frequency are often referred to as a given haplotype. Haplotypes help identify other polymorphic sites on the same chromosome and are implicated in the pathogenesis of certain genetically linked disorders. There are more than 400 genes that encode human ion channels, and an almost endless number of permutations of mutations can occur.

SNPs in various genes have been shown to have relevance for pain sensation, either decreasing or increasing pain perception. Various ion channels are involved in many aspects of in pain signal transduction, transmission, and modulation. Haplotypes of the ion channel genes SCN9A (sodium channel), CACNA2D3 (calcium channel), KCNS1 (potassium channel), CACNG2 (calcium channel), and P2RX7 (ATP-gated ionotropic receptor) have been associated with altered pain sensation postoperatively.^25^ Catecholamine-O-methyltransferase is responsible for breaking down adrenergic and dopaminergic substances; SNPs and haplotypes of decreased catecholamine-O-methyltransferase activity are associated with increased pain sensitivity.^26^ Guanosine triphosphate cyclohydrolase haplotypes are similarly associated with decreased pain in both cancer-related and benign (laminectomy-related) pain.^27^ Pain catastrophizing behavior characterized by high levels of anxiety, vulnerability, and negativism has been linked to the presence of a short allele in the promoter region of 5-HTTLPR, a serotonin transport gene.^28^

The voltage-gated sodium ion channel Na_v_1.7 (encoded by SCN9A) is implicated in painful and painless conditions.^25^ This sodium channel subtype is expressed selectively in sensory and autonomic neurons. Mutations that inactivate the SCN9A gene result in both congenital insensitivity to pain and hereditary sensory and autonomic neuropathy type IID.^25^ Gain-of-function mutations in this gene produce syndromes such as inherited erythromelalgia, paroxysmal extreme pain disorder (familial rectal pain), and small-fiber neuropathy.^25^ Other voltage-gated sodium channel subtypes have also been associated with pain disorders, including Na_v_1.8 (SCN10A) with small-fiber neuropathy and Na_v_1.9 (SCN11A) with congenital insensitivity to pain.^25^

To summarize, there are strong implications regarding a patient’s pain experience and analgesic outcomes as directed by his or her genetic code. Enzymatic activity can significantly alter sensation, addictive behaviors, and medication metabolism, complicating a clinical picture. SNPs and other mutations in ion channel genes may lead to channelopathies that may fundamentally alter how a patient experiences and responds to a nociceptive stimulus. Moreover, some patients may suffer from painful conditions entirely caused by a genetic mutation resulting in a defective channel protein. Understanding of opioid metabolism variability has influenced prescription practices, most notably codeine use in pediatric populations. There is little use of genetic testing in practice today; looking to the future, genetic mapping and screening may help provide direction for pharmaceutical management and abuse risk. Gene therapies may represent a means to affect and interact with these complex systems to prevent or treat acute and chronic pain.

## Conclusion

This review has introduced four novel areas of pain research that show promise in developing both a better understanding of pain responses and new target-specific analgesics for persistent pain.

Targeting of persistent pain mechanisms is focused on preventing or altering neural plasticity. Specific receptor or transmitter agents are acting on key steps in the pain cascade. Personalized medicine through genotyping will reduce risk and improve outcomes using genetically compatible opioid targets.

The cascade of pain transmission is becoming ever clearer yet incredibly complex. There are limitations to the current clinical impact of these areas of study; prospective new agents are either not U.S. Food and Drug Administration approved or are not readily available for immediate general use. NGF antagonists require in-depth assessment of risk–benefit ratios before using in patients with osteoarthritis.

## References

[1] Ramos AMT, Atkinson TJ. Analgesics of the future: inside the potential of nerve growth factor antagonists. Pract Pain Manag. 2019;19:55–59.

[2] Schnitzer TJ, Marks JA. A systematic review of the efficacy and general safety of antibodies to NGF in the treatment of OA of the hip or knee. Osteoarthr Cartil. 2015;23:S8–S17. doi:10.1016/j.joca.2014.10.003. 25527221

[3] Zhou LJ, Peng J, Xu YN, Zeng W-J, Zhang J, Wei X, Mai C-L, Lin Z-J, Liu Y, Murugan M. Microglia are indispensable for synaptic plasticity in the spinal dorsal horn and chronic pain. Cell Rep. 2019;27(13):3844–3859.e6. doi:10.1016/j.celrep.2019.05.087. 31242418 PMC7060767

[4] Taves S, Berta T, Chen G, Ji RR. Microglia and spinal cord synaptic plasticity in persistent pain. Neural Plast. 2013;2013:1–10. doi:10.1155/2013/753656. PMC3759269 24024042

[5] Ji RR, Berta T, Nedergaard M. Glia and pain: is chronic pain a gliopathy? Pain. 2013;154(SUPPL.1):S10–S28. doi:10.1016/j.pain.2013.06.022. 23792284 PMC3858488

[6] Tennant F. Glial cell activation and neuroinflammation : how they cause centralized pain. Pract Pain Manag. 2019;14:1–10.

[7] Mei X-P, Xu H, Xie C, et al. Post-injury administration of minocycline: an effective treatment for nerve-injury induced neuropathic pain. Neurosci Res. 2011;70(3):305–12. doi:10.1016/j.neures.2011.03.012. 21515316

[8] Vallejo R. North American Neuromodulation Society (NANS) abstract. Vol. 1. Las Vegas, NV; 2019.

[9] Sato KL, Johanek LM, Sanada LS, Sluka KA. Spinal cord stimulation reduces mechanical hyperalgesia and glial cell activation in animals with neuropathic pain. Anesth Analg. 2014;118(2):464–72. doi:10.1213/ANE.0000000000000047. 24361846 PMC4297213

[10] Yan Y, Zhou XE, Xu HE, Melcher K. Structure and physiological regulation of AMPK. Int J Mol Sci. 2018;19(3534):1–15. doi:10.3390/ijms19113534. PMC6274893 30423971

[11] Price TJ, Dussor G. AMPK: an emerging target for modification of injury-induced pain plasticity. Neurosci Lett. 2013;557:9–18. doi:10.1016/j.neulet.2013.06.060. 23831352 PMC3844111

[12] Das V, Kroin JS, Moric M, McCarthy RJ, Buvanendran A. Antihyperalgesia effect of AMP-activated protein kinase (AMPK) activators in a mouse model of postoperative pain. Reg Anesth Pain Med. 2019;44(8):781–86. doi:10.1136/rapm-2019-100651. 31229963

[13] Richeimer SH, Lee JJ. Genetic testing in pain medicine—the future is coming. Pract Pain Manag. 2016;16:37–42.

[14] Bertilsson L. Geographical/interracial differences in polymorphic drug oxidation. Current state of knowledge of cytochromes P450 (CYP) 2D6 and 2C19. Clin Pharmacokinet. 1995;29(3):192–209. doi:10.2165/00003088-199529030-00005. 8521680

[15] Cai WM, Nikoloff DM, Pan RM, de Leon J, Fanti P, Fairchild M, Koch WH, Wedlund PJ. CYP2D6 genetic variation in healthy adults and psychiatric African-American subjects: implications for clinical practice and genetic testing. Pharmacogenomics J. 2006;6(5):343–50. doi:10.1038/sj.tpj.6500378. 16550211

[16] Voelker R. Children’s deaths linked with postsurgical codeine. JAMA - J Am Med Assoc. 2012;308(10):963. doi:10.1001/2012.jama.11525. 22968872

[17] Bond C, Laforge KS, Tian M, Melia D, Zhang S, Borg L, Gong J, Schluger J, Strong JA, Leal SM. Single-nucleotide polymorphism in the human mu opioid receptor gene alters β-endorphin binding and activity: possible implications for opiate addiction. Proc Natl Acad Sci U S A. 1998;95(16):9608–13. doi:10.1073/pnas.95.16.9608. 9689128 PMC21386

[18] Boswell MV, Elaine Stauble M, Loyd GE, et al. The role of hydromorphone and OPRM1 in postoperative pain relief with hydrocodone. Pain Physician. 2013;16(3):227–36. 23703421

[19] Bart G, Heilig M, LaForge K, Pollak L, Leal SM, Ott J, Kreek MJ. Substantial attributable risk related to a functional mu-opioid receptor gene polymorphism in association with heroin addiction in central Sweden. Mol Psychiatry. 2004;9(6):547–49. doi:10.1016/j.physbeh.2017.03.040. 15037869 PMC6141020

[20] Tsuang M, Lyons M, Meyer J, Doyle T, Eisen SA, Goldberg J, True W, Lin N, Toomey R, Eaves L. Co-occurrence of abuse of different drugs in men: the role of drug-specific and shared vulnerabilities. Arch Gen Psyhiatry. 1998;55(11):967–72. doi:10.1001/archpsyc.55.11.967. 9819064

[21] Hou QF, Li SB. Potential association of DRD2 and DAT1 genetic variation with heroin dependence. Neurosci Lett. 2009;464(2):127–30. doi:10.1016/j.neulet.2009.08.004. 19664686

[22] Lawford BR, Young RMD, Noble EP, Sargent J, Rowell J, Shadforth S, Zhang X, Ritchie T. The D2 dopamine receptor A1 allele and opioid dependence: association with heroin use and response to methadone treatment. Am J Med Genet - Neuropsychiatr Genet. 2000;96(5):592–98. doi:10.1002/1096-8628(20001009)96:5<592::AID-AJMG3>3.0.CO;2-Y. 11054765

[23] Duaux E, Gorwood P, Griffon N, Bourdel M-C, Sautel F, Sokoloff P, Schwartz J-C, Ades J, Lôo H, Poirier M-F. Homozygosity at the dopamine D3 receptor gene is associated with opiate dependence. Mol Psychiatry. 1998;3(4):333–36. doi:10.1038/sj.mp.4000409. 9702742

[24] Demirbilek H, Galcheva S, Vuralli D, Al-Khawaga S, Hussain K. Molecular sciences ion transporters, channelopathies, and glucose disorders. Int J Mol Sci. 2019;20(10):2590. doi:10.3390/ijms20102590. PMC6566632 31137773

[25] Bennett DLH, Woods CG. Painful and painless channelopathies. Lancet Neurol. 2014;13(6):587–99. doi:10.1016/S1474-4422(14)70024-9. 24813307

[26] Diatchenko L, Slade GD, Nackley AG, Bhalang K, Sigurdsson A, Belfer I, Goldman D, Xu K, Shabalina SA, Shagin D. Genetic basis for individual variations in pain perception and the development of a chronic pain condition. Hum Mol Genet. 2005;14(1):135–43. doi:10.1093/hmg/ddi013. 15537663

[27] Tegeder I, Costigan M, Griffin RS, Abele A, Belfer I, Schmidt H, Ehnert C, Nejim J, Marian C, Scholz J. GTP cyclohydrolase and tetrahydrobiopterin regulate pain sensitivity and persistence. Nat Med. 2006;12(11):1269–77. doi:10.1038/nm1490. 17057711

[28] Kuhnen CM, Samanez-Larkin GR, Knutson B. Serotonergic genotypes, neuroticism, and financial choices. PLoS One. 2013;8(1):e54632. doi:10.1371/journal.pone.0054632. 23382929 PMC3559795

